# The Utilization of a New Immunochromatographic Test in Detection of *Helicobacter pylori* Antibody from Maternal and Umbilical Cord Serum

**DOI:** 10.1155/2014/568410

**Published:** 2014-08-07

**Authors:** Fu-Chen Kuo, Chien-Yi Wu, Chao-Hung Kuo, Chia-Fang Wu, Chien-Yu Lu, Yen-Hsu Chen, Chiao-Yun Chen, Yi-Ching Lo, Ming-Tsang Wu, Huang-Ming Hu

**Affiliations:** ^1^Graduate Institute of Public Health, Kaohsiung Medical University, Kaohsiung 807, Taiwan; ^2^Department of Gynecology and Obstetrics, E-Da Hospital, Kaohsiung 824, Taiwan; ^3^School of Medicine, College of Medicine, I-Shou University, Kaohsiung 824, Taiwan; ^4^Department of Pediatrics, E-Da Hospital, I-Shou University, Kaohsiung 824, Taiwan; ^5^Division of Gastroenterology, Department of Internal Medicine, Kaohsiung Medical University Hospital, Kaohsiung 807, Taiwan; ^6^Department of Medicine, College of Medicine, Kaohsiung Medical University, Kaohsiung 807, Taiwan; ^7^Division of Infectious Diseases, Department of Internal Medicine, Kaohsiung Medical University Hospital, Kaohsiung 807, Taiwan; ^8^Department of Radiology, College of Medicine, Kaohsiung Medical University, Kaohsiung 807, Taiwan; ^9^Department of Medical Imaging, Kaohsiung Medical University Hospital, Kaohsiung 807, Taiwan; ^10^Department of Pharmacology, College of Medicine, Kaohsiung Medical University, Kaohsiung 807, Taiwan

## Abstract

*Background*. *Helicobacter pylori* (*H. pylori*) was linked with several extragastrointestinal diseases, including preeclampsia and intrauterine growth restriction of fetus. One of the signals which can be transferred from mother to fetus is the *H. pylori* IgG antibody. * Aims*. We utilized a commercial immunochromatographic kit to detect the antibody in maternal and cord serum. *Methods*. Three hundred and forty-six females were enrolled and the blood samples were collected on antenatal examination and on delivery. The maternal *H. pylori* infection was determined by stool *H. pylori* antigen test. *Results*. One hundred and five females (30.3%) were *H. pylori*-infected, and the prevalence was higher in immigrants (43.5%) than in Taiwanese (28.7%, *P* = 0.058). The sensitivity, specificity, positive predictive value, negative predictive value, and accuracy of the kit were 77.1%, 88.0%, 73.6%, 89.8%, and 84.7%, respectively. This kit also had similar performance in cord serum. Comparing to the maternal result on delivery, this kit offered a consistent performance in antenatal maternal serum (kappa coefficient 0.92) and in cord serum (kappa coefficient 0.88). *Conclusions*. *H. pylori* IgG antibody can be transferred through the placenta into the fetal circulation. However, accuracy of the test kit needs to be evaluated before utilization in screening.

## 1. Introduction

*Helicobacter pylori* (*H. pylori*), a Gram-negative bacteria existing in the stomach, has been linked with many gastrointestinal diseases, such as peptic ulcer disease, gastric adenocarcinoma, and gastric mucosal-associated lymphoid tissue lymphoma [1]. Following the observation from public health studies, however,* H. pylori* was thought to be associated with several extragastrointestinal diseases [2], such as hematological disease (idiopathic thrombocytopenic purpura, unexplained iron deficiency anemia) [3, 4], cardiovascular disease [5], and neurological disorders [6]. Recently, studies focusing on the obstetric field mentioned the possible influence of* H. pylori* infection in pregnant female. The high prevalence of the* H. pylori* was observed in population who had preeclampsia during pregnancy [7]. The specific gastrointestinal symptom, hyperemesis gravidarum, was also linked with this bacterium [8], although the other study showed conflicting result [9]. These observations raise a concern about the necessity of knowing the* H. pylori* infection status for female in gestational age.

There are several methods to detect of* H. pylori* infection. One of them is the urease test using gastric mucosal tissue obtained during gastroendoscopy. Despite being proven that procedure is safe when performing on the pregnant women [10], the general unwillingness, the high cost, the invasiveness of the procedure, and the possible sampling error make it not the ideal choice for screening the* H. pylori* infection during pregnancy. The noninvasive tests include the urea breath test (UBT), the stool antigen test and the serum* H. pylori* IgG antibody test. The latest one is easy to perform during antenatal examination and the existence of the antibody was found to be associated with the intrauterine growth restriction [11]. How the maternal* H. pylori* antibody influences the growth of the fetus is still elusive, but, interestingly, the antibody can be transmitted transplacentally to the fetus [12, 13]. However, the detection of the serological antibody was frustrated because of the inconsistent accuracy caused by several factors, including the different antigen extracts the kit uses and variable* H. pylori* strain in different region [14, 15]. In the present study, we will evaluate the performance of a new immunochromatographic test kit and detect the existence of the* H. pylori* IgG antibody in both maternal and cord serum.

## 2. Materials and Methods

### 2.1. Subjects and Data Collection

This study was carried out according to the principles of the Declaration of Helsinki and was approved by the Institutional Review Board of E-Da Hospital (EMRP-096-092 and EMRP-099-052). Subjects were recruited from mothers who received regular antenatal examinations and/or delivered their babies at department of Gynecology and Obstetrics of E-Da Hospital in southern Taiwan between April 2008 and September 2011. Participation was voluntary. Informed consent was obtained from each subject and personal data regarding demographic characteristics and obstetric history was collected via questionnaire after interviewing with trained interviewers on participation and/or after baby delivery. Those who have history of gastric surgery, peptic ulcer,* H. pylori*-eradication treatment, or antibiotics or proton pump inhibitor prescription within two month before delivery were excluded. Blood samples from mothers were collected on receiving antenatal examination and/or on admission for delivery. After delivery, blood samples were also collected from umbilical cord vessels representing the existence of* H. pylori* antibody in baby's circulation. The heparinized whole blood was centrifuged at 2,000 rpm for 10 min to isolate plasma supernatant. Stool samples from mothers were supplied during hospitalization for baby delivery. Both stool and serum samples were stored under −20°C until utilized.

### 2.2. Serum* H. pylori* IgG Detection

The IgG antibody to* H. pylori* in serum was detected using a commercial immunochromatographic test kit (ASSURE* H. pylori* Rapid Test, MP Biomedicals, USA). The procedure followed the manufacture's protocol. In summary, 25 *μ*L defrozen serum sample were added into the square well at the lower end of the kit. When the sample front moved upstream the viewing window and reached the pink control line labeled “A,” 2 drops of chase buffer were added into the oval well at upper end of the kit. Then pull the “Hp” marked tab until resistance was felt and add 1 drop of chase buffer into the square well. The result was read after 15 minutes by two trained technicians independently. Positive and negative controls were run simultaneously. The results were determined as both technicians had the same interpretation. The one which had invalid test result or discrepant interpretation was retested using a new device.

### 2.3. Stool* H. pylori* Antigen Detection

Another commercial kit (ImmunoCard STAT! HpSA, Meridian bioscience, Cincinnati, OH, USA), based on a lateral flow chromatography technique using monoclonal antibodies, was utilized for detection of* H. pylori* antigens in human stool. The procedure followed the manufacture's protocol. In summary, stool specimen (5-6 mm in diameter) was transferred into diluent vial and mixed with sample diluent. After vortexing for 15 seconds, break the tip of the vial and dispense 4 drops into the round window at the lower end of the device and read the results after 5 minutes. The results were also interpreted independently by two technicians.

### 2.4. Statistical Analysis

Distribution of demographic and clinical characteristics of participants by* H. pylori *status was reported as means (± standard deviations (SD)) or number (frequency) and was analyzed by independent *t*-test, Chi-square test, and Fischer's exact test, whenever appropriate. The sensitivity, specificity, positive predictive value (PPV), and negative predictive value (NPV) of* H. pylori* status in maternal and umbilical cord serum during delivery were presented, using* H. pylori* status in maternal stool specimens as gold standard. The reliability of* H. pylori* status in maternal serum during delivery and before delivery as well as in maternal serum and umbilical cord serum during delivery was compared by Kappa coefficient. All tests were performed by SAS 9.2 statistical software (SAS Institute Inc., Cary, NC); two-sided *P* value less than 0.05 was considered statistically significant.

## 3. Results

Total 346 pregnant women were enrolled. The demographic characteristics were listed in Tables 1 and 2. Based on the result of stool* H. pylori* antigen detection, 105 subjects were infected with* H. pylori* on baby delivery, 241 subjects had negative stool test results, and the overall infection rate was 30.3%. There was no significant difference between these two groups in body mass index (BMI), education, daily habit (alcohol, smoking, and exercise), underline disease (diabetes mellitus (DM) and hypertension), and obstetric characteristics (parity, history of miscarriage, gestation age (GA), prematurity of infant, placenta weight, and gender of infant). The only difference but without significance was the nationality (*P* = 0.058). Within the enrolled subjects, 39 were immigrants from Singapore (2), Thailand (2), Cambodia (3), Vietnam (14), and China (18), respectively. Seventeen (43.5%) belonged to the* H. pylori-*infected group, and the prevalence was higher than in Taiwanese (28.7%).

Next, we evaluated the existence of* H. pylori* IgG antibody in maternal serum. Using the immunochromatographic device to test the serum sample collected on delivery, the sensitivity, specificity, positive predictive value (PPV), negative predictive value (NPV), and accuracy were 77.1%, 88.0%, 73.6%, 89.8%, and 84.7%, respectively (Table 3). This commercial test also had similar performance when testing the serum collected from umbilical vein. Within 338 umbilical serum samples (100 from babies delivered from* H. pylori*-infected mothers), the sensitivity, specificity, and accuracy were 69.0%, 91.6%, and 84.9%, respectively, in comparison with maternal stool test results.

This commercial test also offers consistent results (Table 4). Within 324 participants who donated two blood samples (one collected when receiving antenatal examination and the other collected during admission for delivery), 314 had consistent result. Two participants were positive in antenatal test but negative in delivery. The situation of the other 8 cases was reversed, with negative result on antenatal but positive on delivery. The kappa coefficient was 0.92 (95% CI 0.88–0.97). Besides, when comparing between the maternal (on delivery) and the cord serum, 89 out of 105* H. pylori*-infected mothers had positive cord serum antibody detection from their babies. On the other hand, none of the baby's cord serum from* H. pylori*-negative mothers showed the existence of* H. pylori *antibody. The kappa coefficient was 0.88 (95% CI 0.83–0.93).

## 4. Discussion 

In the present study, we evaluated the* H. pylori* IgG antibody from maternal and cord serum using a commercial immunochromatographic test kit. It has been shown that the human immune system can produce variable antibodies with different molecular weight against* H. pylori*, either in serum (IgG) or in human milk (IgA) [13]. We found that, in cord serum, there was detectable* H. pylori* IgG antibody, and all these seropositive babies were delivered from seropositive mothers. The consistency of the* H. pylori* IgG antibody detection between maternal and cord serum suggested that the antibody in cord serum was transferred transplacentally, similar to the findings reported previously [12, 13]. However, the antibody was not detected from 16 infants who were delivered from* H. pylori*-seropositive mothers, and the relatively low antibody titer in cord serum might be the reason.

We utilized the stool antigen test as the gold standard of* H. pylori* infection. In fact, both UBT and stool antigen test are acceptable noninvasive test [16]. UBT has better performance, with a sensitivity of 88–95% and a specificity of 95%–100% [17]. It has also been proven that both 13C- (nonradioactive) and 14C-UBT (radioactive) are harmless in the pregnant female [18], with the possibility of the fetal radiation dose in the latter being much lower than the dose considered teratogenic [19]. However, the high cost and the unavailability in clinic limit its utilization. In the present study, we chose the stool antigen test for determining the* H. pylori* status and this test has been shown to have equal performance to UBT, with a sensitivity of 94% and a specificity of 92% [20].

Detection of the* H. pylori* IgG antibody in serum is an alternative method. The advantage of this test is that it is not affected by local changes in the stomach, such as bleeding, that could lead to false-negative results in the other tests. For female in pregnancy, it is an option to screen when they receive the regular antenatal examinations, using commercial available test kit. In the present study, we confirmed that the results obtained antenatally were consistent with the ones checked on baby delivery. However, this method has several disadvantages. Firstly, the performance of each test kit varies in different regions [21]. This depends on the* H. pylori* strain chosen for development of the IgG antibody and the prevalence of the strain in the given region, as the* H. pylori* strain differs in different countries or area [22]. Therefore, it is necessary to know the performance of a new test kit before utilizing it for screening. In the present study performed in southern Taiwan, the sensitivity, specificity, and accuracy of this test kit were 77.1%, 88.0%, and 84.7%, respectively, relatively lower than the data in manufacturer's instructions which was tested in the other Asian countries. Secondary, the result cannot represent the current infection, as the antibody is still detectable months to years after successful* H. pylori* eradication [23, 24]. So it raises a concern about the interpretation of the association between disease and* H. pylori* when the infection is determined by the existence of* H. pylori* IgG antibody. All mentioned above might be the reasons to cause fluctuated association between* H. pylori* and variable disease, including preeclampsia [25–27].

It has been mentioned that maternal* H. pylori* infection influences the development of the fetus [11]. The mechanism of intrauterine growth restriction is still elusive. Previous study proved that transmission of infection from mother to infant was not detected on delivery [28]. Therefore, the other factors associated with this bacterium, such as the transplacentally acquired antibody, should be taken into consideration. In fact, the human immune system responding to the* H. pylori* produces variable antibodies, and most of them can be detected in cord serum [13]. Although it has been shown that the transplacentally acquired antibody does not protect infants from colonization by* H. pylori *[12], it is necessary to further investigate its possible role during pregnancy.

Following the improvement of the hygiene condition and the comprehensive utilization of eradication, the prevalence of* H. pylori* infection is decreasing in Taiwan. The overall infection rate in the present study was 30.3%. Interestingly, when classifying the* H. pylori*-positive group, higher infection rate (43.5%) was found in immigrants who came from China and south-east Asian countries where the* H. pylori* prevalence was high [29]. It is worth observing how this situation will influence the prevalence of* H. pylori* in their next generation.

## 5. Conclusion

*H. pylori* is not only connected with gastrointestinal symptoms; in pregnant female, the related antibody can transfer through the placenta into the fetal circulation. The IgG antibody can be detected during antenatal examination using commercial test kit. However, accuracy of the test kit needs to be evaluated before utilization in screening. And the possible role of the* H. pylori* IgG antibody in the fetal development needs further investigation.

## Figures and Tables

**Table 1:** Demographic and clinical characteristics of participants who were subgrouped according to *H. pylori* status determined by stool antigen test (HpSA) on delivery (*N* = 346).

	*H. pylori* status determined by HpSA				*P* value
	*H. pylori* (+)		*H. pylori* (−)		
	*n*	Mean ± SD	*n*	Mean ± SD
Age (yrs)	105	29.24 ± 4.52	241	29.15 ± 4.49	0.872
Height (cm)	105	157.99 ± 5.04	241	158.88 ± 4.92	0.124
Prepregnant weight (kg)	102	53.94 ± 7.84	237	55.63 ± 9.61	0.119
Prepregnant BMI	102	21.58 ± 2.79	237	22.03 ± 3.64	0.209
Weight gain (kg)	102	13.46 ± 4.02	237	13.02 ± 5.07	0.436
Placenta weight (g)	105	652.09 ± 126.10	240	645.42 ± 152.05	0.694
GA (weeks)	105	38.57 ± 1.17	241	38.45 ± 1.42	0.451

SD: standard deviation.

**Table 2:** The distribution of demographic and clinical characteristics of participants according to the *H. pylori* status determined by stool antigen test (HpSA) on delivery (*N* = 346).

	*H. pyloric* status determined by HpSA		
	*H. pylori* (+)	*H. pylori* (−)	*P*-value
	*n* (%)	*n* (%)	
Education			
<college	48 (45.7)	90 (37.5)	0.152
≧college	57 (54.3)	150 (62.5)	
Nationality			
Taiwan	88 (83.8)	218 (90.8)	0.058
Immigrant	17 (16.2)	22 (9.2)	
Smoking status			
Yes	2 (1.9)	8 (3.3)	0.729^a^
No	103 (98.1)	233 (96.7)	
Alcohol drinking			
Yes	0	2 (0.8)	1.000^a^
No	105 (100.0)	238 (99.2)	
Exercise			
≧1 times/week	37 (35.2)	69 (28.8)	0.229
<1 times/week	68 (64.8)	171 (71.3)	
Diabetes mellitus			
Yes	4 (3.8)	8 (3.3)	0.760^a^
No	101 (96.2)	233 (96.7)	
Hypertension			
Yes	3 (2.9)	17 (7.1)	0.124
No	102 (97.1)	224 (92.9)	
Parity			
1	51 (48.6)	121 (50.2)	0.806
2	43 (41.0)	100 (41.5)	
>3	11 (10.5)	20 (8.3)	
Miscarriage			
Yes	13 (12.4)	32 (13.3)	0.820
No	92 (87.6)	209 (86.7)	
Type of delivery			
NSD/VED	72 (68.6)	158 (65.6)	0.585
C/S	33 (31.4)	83 (34.4)	
Prematurity			
<37 weeks	4 (3.8)	11 (4.6)	1.000^a^
≧37 weeks	101 (96.2)	230 (95.4)	
Baby gender			
Male	58 (55.2)	140 (58.1)	0.622
Female	47 (44.8)	101 (41.9)	

^a^Fisher's exact test.

**Table 3:** The performance of the immunochromatographic test kit (ASSURE *H. pylori* Rapid Test) in detection of *H. pylori* IgG antibody in maternal serum collected on delivery and cord serum in comparison to the stool antigen test.

	Sensitivity	Specificity	PPV	NPV	Accuracy
	%	%	%	%	%
	(*n/N*)	(*n/N*)	(*n/N*)	(*n/N*)	(*n/N*)
Maternal serum	77.1	88.0	73.6	89.8	84.7
	(81/105)	(212/241)	(81/110)	(212/236)	(293/346)
Umbilical serum	69.0	91.6	77.5	87.6	84.9
	(69/100)	(218/238)	(69/89)	(218/249)	(287/338)

*n*: serum sample number; *N*: stool sample number.

**Table 4:** The consistent performance of the immunochromatographic test kit (ASSURE *H. pylori* Rapid Test) in detection of *H. pylori* IgG antibody in cord serum and maternal serum collected antenatally and on delivery.

	Maternal serum on delivery		Kappa coefficient
	*H. pylori* (+)	*H. pylori *(−)	(95% CI)
Maternal serum, antenatal			
*H. pylori* (+)	93	2	0.92
*H. pylori* (−)	8	221	(0.88–0.97)
Cord serum			
*H. pylori* (+)	89	0	0.88
*H. pylori *(−)	16	233	(0.83–0.93)
